# Incidental Hemangioma in Tubectomy and Hemorrhoidectomy Specimens: A Report of Two Cases

**DOI:** 10.7759/cureus.64390

**Published:** 2024-07-12

**Authors:** Sneha Jawalkar, Kezia A Jacob, Vijayalaxmi S Patil

**Affiliations:** 1 Pathology and Laboratory Medicine, Shri B M Patil Medical College, Hospital and Research Centre, Bijapur Lingayat District Educational (BLDE) (Deemed to be University), Vijayapura, IND; 2 Pathology, Shri B M Patil Medical College, Hospital and Research Centre, Bijapur Lingayat District Educational (BLDE) (Deemed to be University), Vijayapura, IND

**Keywords:** cd34+, cavernous, vascular tumors, hemorrhoids, anal mucosa, fallopian tube, hemangioma

## Abstract

Benign vascular neoplasms, or hemangiomas, can develop anywhere in the body.As they are usually asymptomatic, they are discovered incidentally while evaluating other coexisting diseases or conditions. We herein report two cases of capillary hemangioma at two extremely rare sites. A woman in her early 30s with a history of nine months of amenorrhea came for safe confinement and underwent an elective lower segment cesarean section (LSCS) with bilateral concurrent tubectomy. Another case involved a man in his 40s who presented with bleeding per rectum for three months. Per rectal examination, two purplish red masses were noted at the 3 and 11 o'clock positions, which were noncompressible and did not bleed on touch. Subsequently, a hemorrhoidectomy was performed. A well-defined vascular lesion in the fallopian tube and hemorrhoidal tissue were seen during the histopathological examination, which was compatible with a capillary hemangioma. The vascular endothelium was emphasized by immunostaining with CD34.Due to the potential for these lesions to manifest as surgical emergencies, it is imperative for surgeons to recognize and appropriately manage such presentations.

## Introduction

Hemangiomas are benign vascular lesions that manifest as an overgrowth of blood vessels, often appearing on the skin or internal organs [1]. Since hemangiomas are typically asymptomatic, they are frequently discovered incidentally during evaluations for other conditions. Hemangiomas, benign tumors originating from endothelial cells, can manifest at any age, from infancy to adulthood [2]. Fallopian tube hemangiomas occur in 1.1% to 1.7% of cases, whereas intestinal hemangiomas account for only approximately 0.05% of all gastrointestinal tumors, with the small intestine being the most common site and the colon being the second most common site in the gastrointestinal (GI) tract [1].

Although they commonly occur on the skin, subcutaneous tissue, and oral mucosa, they rarely affect internal organs, particularly the female genital tract [3]. According to Ragins et al., the first cavernous hemangioma of the fallopian tube was reported in 1947. Even though they are found incidentally, hemangioma of the fallopian tube may present with abdominal pain or dysmenorrhea [4].

The rectosigmoid colon is typically affected by cavernous hemangiomas [5]. Rectal and anal hemangiomas are very vascular, bleeding is a severe complication, and approximately 80% of the time, they are misdiagnosed because they resemble more prevalent conditions such as internal hemorrhoids, inflammatory bowel disease, or colon cancer that all cause bleeding in the lower GI tract. In this report, two cases of capillary hemangioma involving the fallopian tube and anal mucosa are presented.

## Case presentation

Case 1

A woman in her early 30s with Gravida 3, Para 2, Live 2, and a history of nine months of amenorrhea came for safe confinement. She underwent an elective lower segment cesarean section (LSCS) with bilateral concurrent tubectomy. Her prenatal course was uneventful.

**Figure 1:**
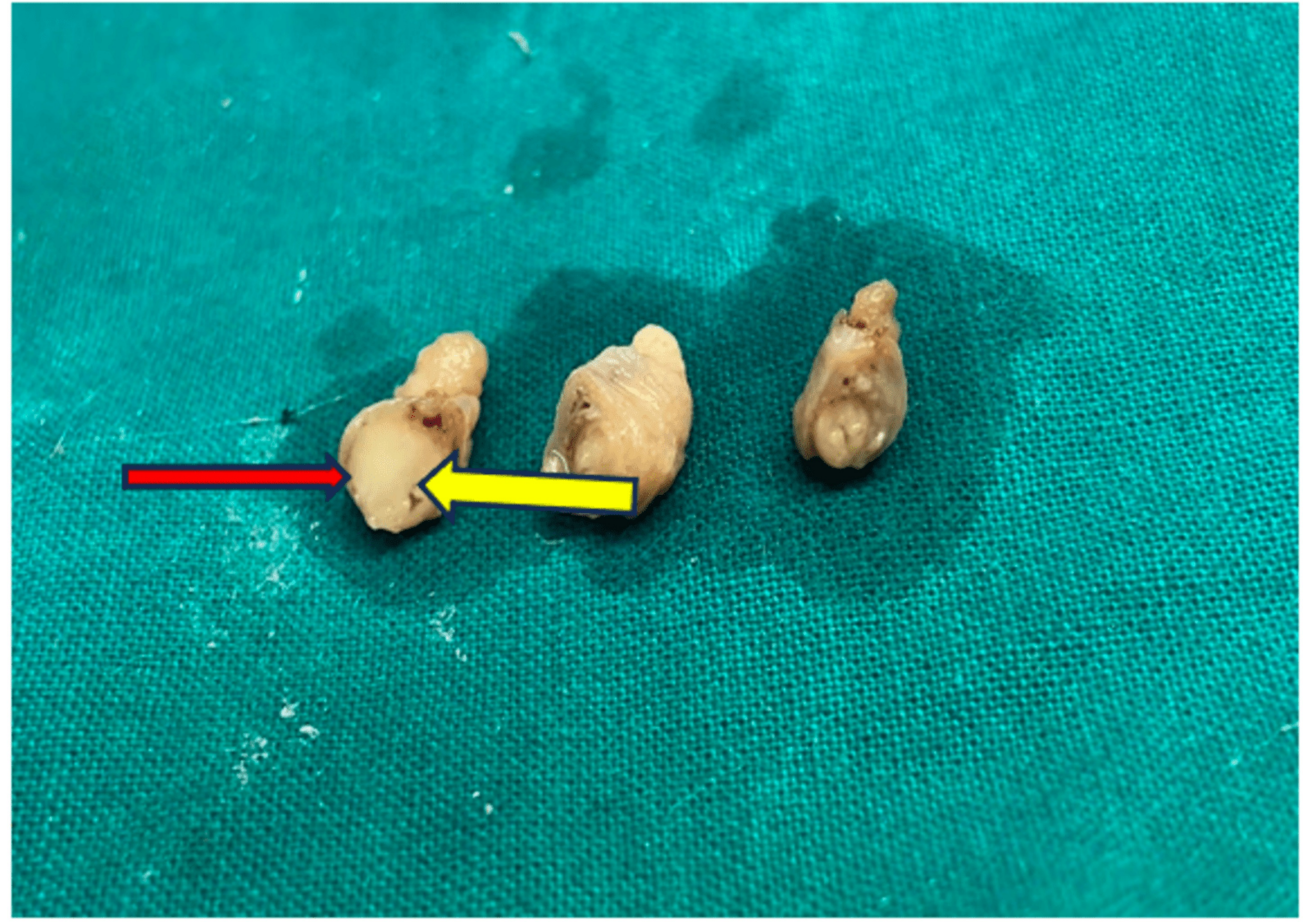
Case 1: the fallopian tube measuring 1 cm in length. A cut section of the fallopian tube showing a small gray-white solid nodule in the wall of the tube (red arrow) with the lumen pushed to the periphery (yellow arrow).

The tubectomy specimens were sent for histological confirmation. Macroscopically, the two tubular structures measured 1 cm in length. A cut section of one of the tubes showed an eccentrically pushed narrow lumen, and the tube wall showed a gray-white, well-circumscribed, homogenous solid nodule measuring 2 mm in diameter (Figure 1).

**Figure 2:**
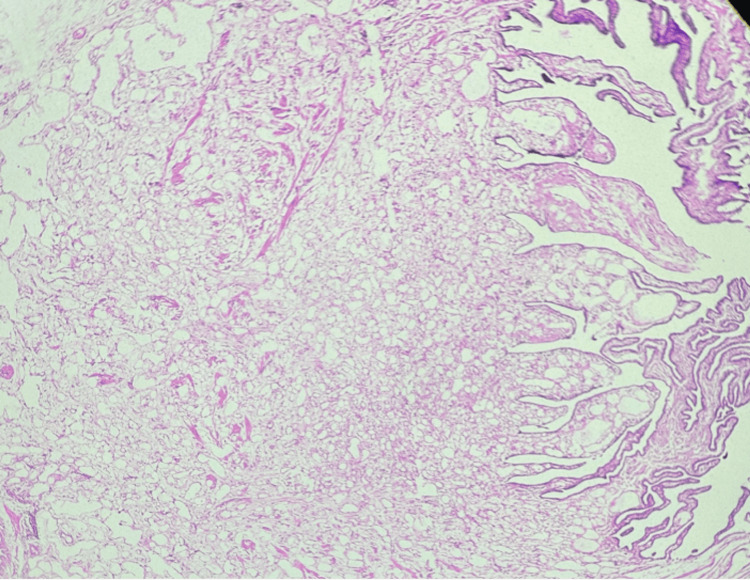
Case 1: photomicrograph (100×, hematoxylin & eosin stain) showing a normal fallopian tube mucosa on the right side. The left side shows a lesion comprised of closely packed capillaries in the wall of the tube, suggestive of a capillary hemangioma.

Microscopically, a well-defined and unencapsulated tumor tissue was observed in the muscle layer of the tube, consisting of capillary-sized vascular channel lobules lined by a single layer of flattened endothelial cells in conjunction with the histology of normal fallopian tubes (Figure 2). No evidence of necrosis or mitotic activity was seen within the tumor.

**Figure 3:**
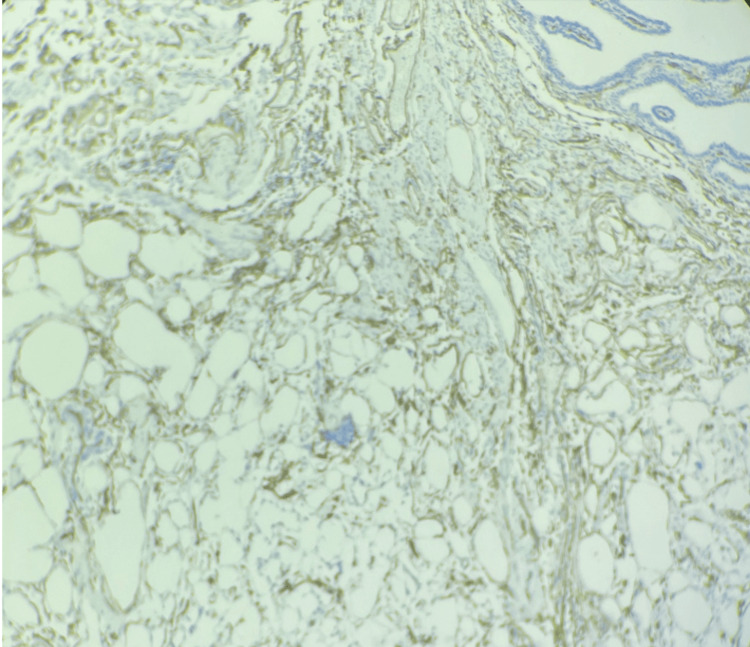
Case 1: photomicrograph (200×) immunohistochemical study of CD34. The endothelial cell lining of capillaries shows immunopositivity.

A photomicrograph of an immunohistochemistry slide shows a diffuse CD34 positivity in the endothelial cells (Figure 3).

Case 2

A man in his early 40s presented with bleeding per rectum and constipation for three months. There is no history of weight loss, loss of appetite, abdominal pain, or mass per abdomen.

Per rectal examination, two purplish red masses were noted at the 3 and 11 o'clock positions, which were noncompressible and did not bleed on touch. Clinically, it was diagnosed as hemorrhoids. Subsequently, a hemorrhoidectomy was performed, and the specimen was sent for histopathological examination.

Grossly, there were multiple dark brown tissue bits measuring between 1.2 and 0.5 cm. On the cut section, one of the bits showed a circumscribed pale brown solid nodule measuring 1x1 cm.

**Figure 4:**
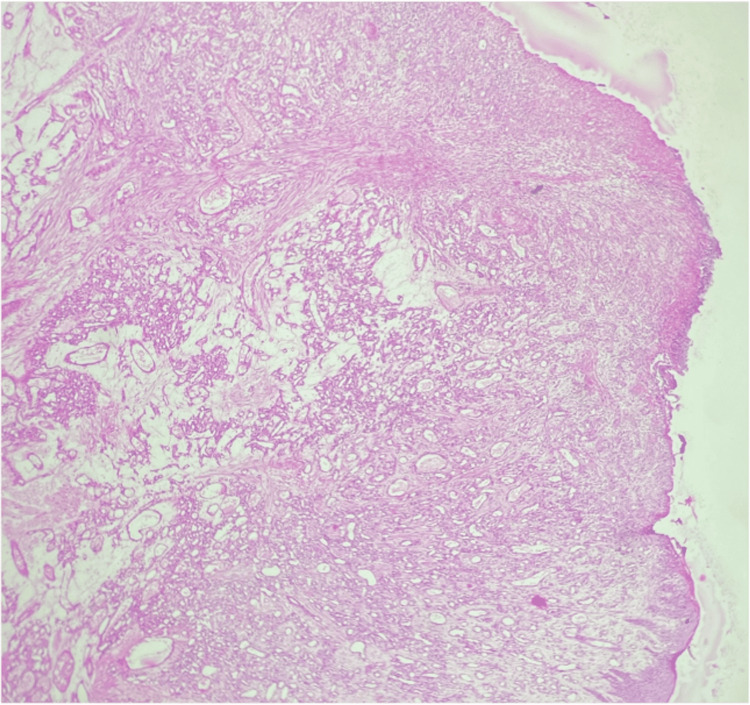
Case 2: photomicrograph (40×, hematoxylin & eosin stain) showing ulcerated lining epithelium. The subepithelium shows lobules of closely packed capillaries, suggestive of a lobular capillary hemangioma.

Microscopically (Figure 4), hemorrhoidal tissue of the anorectal lining epithelium with thrombosed blood vessels. A well-circumscribed, unencapsulated tumor tissue lined by stratified squamous epithelium was also noted, showing areas of ulceration and inflammation. The tumor tissue was arranged in lobules separated by fibrous septa. Variably sized capillary vascular channels with a single layer of endothelial cells lining them closely comprise the lobules. The endothelium-lining cells showed no signs of necrosis or mitotic activity. Histopathologically, it was diagnosed as lobular capillary hemangioma.

**Figure 5:**
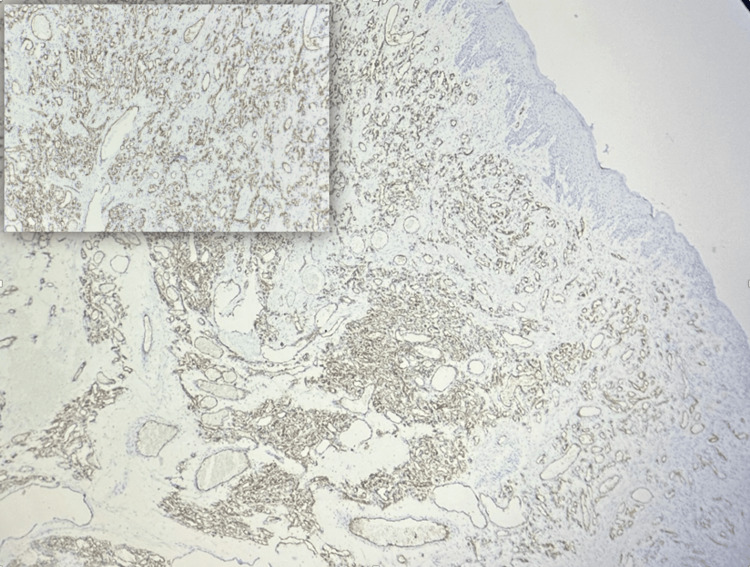
Case 2: photomicrograph (40×): an immunohistochemical study showing CD34 immunopositivity in the endothelial cells lining the capillaries. The inset shows a higher magnification (100×).

An immunohistochemistry analysis was conducted, and the tumor showed positivity for the endothelial cell marker CD34, thus confirming the pathological diagnosis (Figure 5).

Lymphangioma, vascular leiomyoma, mesothelioma, and adenomatoid tumors are the histopathological differentials of these cases, which were ruled out after immunohistochemical analysis [6].

## Discussion

Tumors of the fallopian tube are rare, with an incidence rate significantly lower than other neoplasms affecting the female genital tract. The common neoplasms in the fallopian tube are adenomatoid tumors, papillary cystadenomas, mucinous lesions of the fallopian tube, leiomyoma, and adenofibromas. Hemangioma of the fallopian tube is a rare benign vascular tumor that occurs sporadically. Typically, these tumors range in size from 2 to 10 mm, do not cause symptoms, and are frequently found accidentally during surgeries for other reasons. The etiology remains unclear. Fallopian tube hemangioma is associated with the effects of estrogens, which stimulate the growth of blood vessels. Twelve prior reports of fallopian tube hemangiomas in patients aged 13 to 77 have been found in the published literature, out of which only two cases were of capillary hemangioma, and the remaining all were of cavernous hemangioma [2]. Typically, anemia and a history of painless, recurring rectal bleeding are present in cases of intestinal hemangiomas [5].

Hemangiomas can occur singly or in multiples. Multiple hemangiomas are frequently linked to related lesions on the skin or in other organs, such as the liver. Additionally, they may manifest as a constituent of syndromes such as Maffuci syndrome, Osler-Weber-Rendu syndrome, Klippel-Trenaunay-Weber syndrome, and blue rubber bleb nevus syndrome [1].

Hemangiomas usually appear on ultrasound as well-defined, echogenic lesions, whereas CT scans typically show hypodense areas with sustained contrast enhancement. MRI imaging generally shows that hemangiomas are well-defined lesions with substantial signal intensity on T2-weighted images and persistent enhancement on delayed images [7].

Cavernous hemangiomas appear on colonoscopy as a single or cluster of soft, red, blue, or submucosal lesions that are easily compressed during insufflation and have engorged veins around them in the rectal wall [5]. A biopsy is not advised because of the lesion’s vascular nature.

In a study conducted by Ebrahimi et al., a patient who underwent surgery for endometrial adenocarcinoma presented with a slight bulge in the midportion of the fallopian tube, which histologically was confirmed to be a cavernous hemangioma [4]. The authors suggested a potential association between hormonal therapy and the development of hemangiomas, particularly during menstruation or pregnancy.

Patel et al. also reported a case in which a patient with discomfort in the right lower quadrant of the abdomen was first diagnosed with acute appendicitis; however, subsequent investigations revealed hemoperitoneum and a right tubal mass. Histological examination confirmed the diagnosis of cavernous hemangioma [4].

Hemangiomas have a smooth or tuberous surface and appear as cyanotic or red nodes when viewed macroscopically. In imaging studies, they typically present as well-defined hypervascular masses with characteristic enhancement patterns [1]. Histologically, hemangiomas comprise constantly growing capillaries, eventually forming canaliculi with a single layer of endothelium lining them. The endothelium lining the vascular channels is highlighted by immunostaining for CD34. Hemangiomas show positivity for CD34, CD31, vimentin, and smooth muscle actin. Ki67 helps to differentiate benign from malignant vascular lesions [4].

A benign condition of the submucosal vascular system, colonic hemangioma usually has a good prognosis if the lesion is completely removed. Hemangiomas have a favorable prognosis, and there is complete involution in most cases.

In 2017, a study conducted by Bean et al. revealed the recurrence of *GNAQ* mutations in anastomosing hemangiomas. The role of *GNAQ* mutations helps distinguish low-grade angiosarcoma from anastomosing hemangiomas [8].

Hemangiomas can undergo infarction, thrombosis, fibrosis, and other complications that may impact their clinical course [7]. Larger lesions may manifest as hemoperitoneum from a ruptured hemangioma, a torsion of the ovary, or an acute abdomen.

Smaller submucosal hemangiomas can be endoscopically removed, while larger and more diffuse rectosigmoid hemangiomas are typically managed surgically [1-5]. Both upper and lower GI endoscopies can be performed with laparoscopy for comprehensive evaluation and treatment planning [1]. This is a safer method for the resection of duodenal hemangiomas. The laparoscopic approach allows for an early recovery of bowel function and reduces the need for postoperative analgesics by avoiding open surgery.

Surgical management of hemorrhoids is deemed more effective than alternative conservative therapeutic approaches, given that the requirement for retreatment is estimated to range from 0% to 20%. The selection of appropriate surgical treatment for hemorrhoids necessitates an assessment of the effectiveness and risks associated with the technique, weighed against the potential benefits for the patient [9,10].

## Conclusions

When detected incidentally, hemangiomas have to be reported, even if the lesion is very tiny. This allows the surgeon to be watchful about any excessive, uncontrollable bleeding from surgical sites in the postoperative period and further evaluate the patient for any vascular lesions at other sites. Hemangiomas tend to have a good prognosis and are cured by complete excision. It is crucial that endoscopists and surgeons take this lesion into account when making a differential diagnosis of gastrointestinal bleeding.
